# La mort subite de l’adulte, particularités en Afrique, à propos de 476 cas

**DOI:** 10.11604/pamj.2013.16.125.2490

**Published:** 2013-11-29

**Authors:** Mohamed Maniboliot Soumah, Drissa Kanikomo, Mor Ndiaye, Mamadou Lamine Sow

**Affiliations:** 1 Service de Médecine Légale, B.P. 7080, Faculté de Médecine, Pharmacie et Odontostomatologie, Université Cheikh Anta Diop (UCAD), Dakar, Sénégal

**Keywords:** Mort subite, cardiopathies, autopsie, Sudden death, cardiopathies, autopsy

## Abstract

La mort subite est une mort instantanée ou rapide, d’origine naturelle, inattendue ou inopinée. Chez l’adulte les causes cardiovasculaires, pulmonaires et cérébrales prédominent. Son mode de survenue est dramatique. L’objectif était ici de déterminer les circonstances de survenue de ces morts subites, les facteurs de risque, d’identifier les causes de mort subite chez l’adulte à l’autopsie, en vue d’améliorer la prévention. Il s’agit d’une étude rétrospective portant sur les cas de morts subites ayant fait l’objet d’une autopsie au service d’anatomie pathologique de l’Hôpital Aristide Le Dantec (HALD) de Dakar du 1er janvier 2003 au 31 décembre 2005. Durant cette période 476 cas de morts subites chez l’adulte sur 1936 autopsies ont été enregistrés. Le sexe ratio est de 2,9 avec 356 hommes (74,8%) et 120 femmes (25,2%). Les cardiopathies ont été la première cause de décès dans notre série avec 177cas sur 476 soit 37,2%. La prévention de la mort subite reste primordiale, surtout dans le contexte africain, où la prise en charge pré hospitalière est souvent défaillante.

## Introduction

“La mort subite est une mort naturelle qui survient de façon inopinée chez un sujet en bon état de santé apparent dans un délai de moins d’une heure après l’apparition de symptômes éventuels, c′est-à-dire après une agonie brève” [1]. Chez l’adulte les causes cardiovasculaires, pulmonaires et cérébrales prédominent. Plusieurs facteurs (HTA, obésité, hypercholestérolémies, tabagisme, troubles du rythme cardiaque, sédentarité) sont fréquemment cités dans la littérature [2–5]. Bien que la mort subite chez l’adulte ne soit pas exceptionnelle en Afrique, les publications en la matière sont rares. Nous avons effectué ce travail dans le but de déterminer les causes de mort subite en vue d’améliorer la prévention. Les objectifs de ce travail sont aussi de déterminer les circonstances de survenue de ces morts subites, les facteurs de risque et d’identifier les causes de mort subite chez l’adulte, âgé de plus de 18 ans.

## Méthodes

Cette étude rétrospective porte sur les cas de morts subites ayant fait l’objet d’une autopsie au service d’anatomie pathologique de l’Hôpital Aristide Le Dantec (HALD) de Dakar du 1er Janvier 2003 au 31 Décembre 2005. Durant cette période 476 cas de morts subites chez l’adulte sur 1936 autopsies ont été enregistrés. Les documents étudiés étaient les réquisitions des autorités policières ou de la gendarmerie, le registre d’autopsie, les rapports d’autopsie.

## Résultats

**Fréquence:** durant les 3 années d’étude, 476 cas de mort subite chez l’adulte, ont été enregistrés, soit 158,7 cas par an avec une augmentation de 2003 à 2005. Les 476 cas de mort subite représentaient 24,6% des autopsies réalisées durant la même période. Les morts subites constituent le deuxième motif d’autopsie (Tableau 1), après les accidents de la circulation (36,2%).

**Tableau 1:** Place de la mort subite dans les motifs d’autopsie

Motifs	2003	2004	2005	Total
Mort subite adulte	133	164	179	476
Accident voie publique	315	245	198	758
Noyade	76	54	70	200
Coups et blessures	52	50	36	138
Accident de travail	11	13	16	40
Suicide	7	12	10	29
Brulure	5	10	12	27
Electrocution	8	6	9	23
Infanticide	3	7	9	19
Pendaison	3	3	6	12
Autres	67	75	72	214
**Total**	**679**	**640**	**617**	**1936**

**Figure 1:**
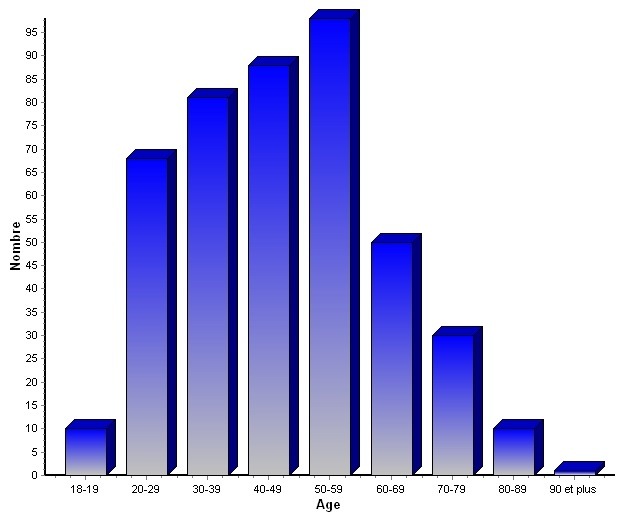
Répartition des morts subites selon l’âge

**Répartition selon l’âge et le sexe:** l’âge moyen des patients dans notre série est de 45,5 ans avec des extrêmes allant de 18 à 90 ans. Dans notre série le maximum de cas est enregistré entre 50 et 55 ans (Figure 1). Le sexe ratio est de 2,97 avec 356 hommes (74,8%) et 120 femmes (25,2%).

**Facteurs de risque:** l’obésité, le facteur le plus fréquent, a été notée dans 51 cas.

**Figure 2:**
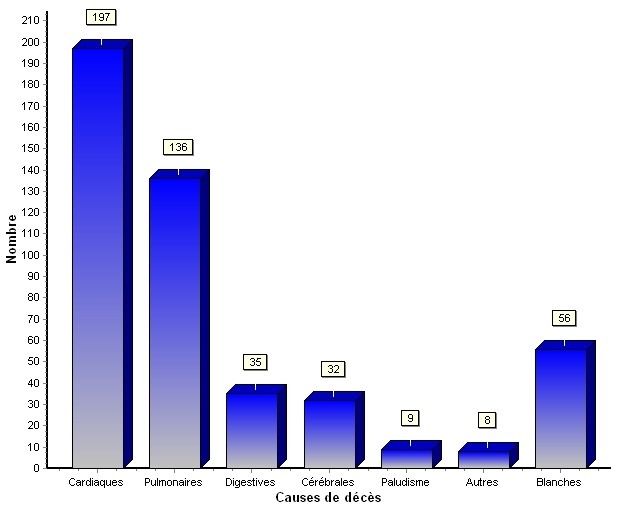
Répartition des morts subites selon la cause

**Causes de décès:** les causes cardiovasculaires hors atteinte cervicocéphalique ont été la première cause de décès dans notre série avec 197 cas sur 476 soit 41,4% (Figure 2). Elles sont suivies par les affections pulmonaires 136 cas soit 28,5%, digestives et des organes annexes 35 cas (7,3%) et cérébrales 32 cas (6,7%).

**Les causes cardiaques** sont dominées par les infarctus du myocarde et la thrombose coronarienne, soit 48,7% des causes cardiaques et 20,1% de l’ensemble des morts subites. Les cardiomyopathies (21,3%) et les endocardites (20,8%) représentent respectivement la deuxième et la troisième cause cardiaque. Quant aux valvulopathies, retenues responsables de 3% des causes cardiaques, elles sont associées à d’autres cardiopathies ou à des pneumopathies dans 45 cas soit 9,4% de nos cas. On observe une augmentation des morts subites par cause cardiaque d’année en année; cette augmentation se fait surtout sur les infarctus du myocarde qui représentent sur les 3 années d’étude, 16,5% des causes cardiaques puis 21,3% et enfin 21,7% des causes cardiaques de mort subite (Tableau 2).

**Tableau 2:** Répartition des causes cardiovasculaires hors atteinte cervicocéphalique

Affections cardiaques	2003	2004	2005	Total
Infarctus du myocarde	22	35	39	96
Myocardiopathie	11	15	16	42
Endocardite	12	9	20	41
Valvulopathie	3	1	2	6
Péricardite	1	2	3	6
Rupture anévrysme aortique	2	3	1	6
**Total**	**51**	**65**	**81**	**197**

**Les causes pulmonaires** sont dominées par les broncho-pneumopathies (36%), suivies par l’embolie pulmonaire et l’œdème pulmonaire aigu respectivement (25% et 14,7%). La tuberculose a été retenue comme cause dans quatre cas sans confirmation bactériologique.

**Les causes digestives:** les hémoperitoines constituent 40% des causes digestives suivis par les péritonites et les abcès avec 34,2%.

**Les causes cérébrales** sont les hémorragies intracrâniennes dans 71,8% des cas. Elles se composent de 12 hémorragies cérébro-meningées, 10 cas hématomes sous duraux chroniques, un anévrysme rompu. L’œdème cérébral occupe la 2e place avec 12% des décès par atteinte cérébrale.

**Les autres causes** sont le paludisme (9 cas), une tumeur pancréatique (2 cas), la pyélonéphrite (4 cas), une tumeur rénale (1 cas), une hydronéphrose (1 cas), une tumeur rétro péritonéale (1 cas).

## Discussion

La mort subite de l’adulte demeure un problème dans tous les pays, quelque soit le niveau de développement. Nous notons 476 cas en 3 ans soit une moyenne de 158,7 cas par an soit 1,58/100 000 habitants. En Europe, la fréquence des morts subites est de 1/100 000 habitants pour les adultes jeunes et de 1 à 2/1000 habitants chez les adultes de plus de 30 ans avec un pic entre 45 et 75 ans [2]. En Côte d’Ivoire, Yappo Etté H [5] retrouvait 171 cas sur 2 ans soit en moyenne 85,5 cas par an et 1/200 000 habitants.

L’âge moyen dans notre série est de 45,5 ans avec un maximum de cas entre 50 et 55 ans. Le sexe ratio est de 2,97. Le contraire est retrouvé en Côte d’Ivoire [5] avec un pic de fréquence entre 18 et 29 ans et entre 30 et 39 ans. Une nette régression apparaissait à partir de 61 ans. Dans les pays développés la mort subite survient surtout chez la personne âgée avec un âge moyen de 68 ± 20 ans [3, 6], 65ans chez l’homme et 77 ans pour la femme [4]. La mort subite est plus fréquente chez l’homme [3–6] dans toutes les séries.

Les causes cardiovasculaires sont première cause de mort subite dans toutes les séries. Dans les pays occidentaux, la fréquence des morts subites d’origine cardiaque est de 88% des morts subites pour les adultes d’âge moyen et les sujets âgés. En Europe et dans les pays développés en général la mortalité était liée aux complications de l’athérosclérose dans les années 70. La prévention des facteurs de risque d’ordre nutritio-métabolique a permis une baisse de cette mortalité [7]. Des affections spécifiques sont individualisées, ce sont: le syndrome de Brugada, le syndrome du QT court familial, le syndrome de Wolff-Parkinson-White qu’il soit symptomatique ou non, le prolapsus valvulaire mitral. Pour ces affections, l’autopsie n’apporte pas de renseignement. Les valvulopathies rhumatismales ont aussi progressivement disparu ainsi que les endocardites qui se développaient sur ces dernières. Les seuls facteurs prédisposant de ces endocardites en Europe restent la toxicomanie intraveineuse, les prothèses valvulaires, les scléroses valvulaires dégénératives liées au vieillissement et les infections nosocomiales [8]. La cardiomyopathie ventriculaire droite arythmogène (CVDA) est retrouvée parmi les causes de mort subite en Europe et la mort subite est la première manifestation de cette pathologie. C’est une entité anatomo-clinique polymorphe caractérisée par une infiltration adipeuse du myocarde avec persistance de fibres myocardiques survivantes entourées de fibrose [9]. A l’autopsie le diagnostic peut être suspecté par la découverte d’une dilatation ventriculaire droite localisée ou diffuse, mais seule la microscopie par la mise en évidence de cardiomyocytes disposés en travées parallèles, engainés d’un tissu collagène dense au sein du tissu adipeux [10] pourra affirmer le diagnostic. La cardiomyopathie la plus fréquemment associée à la mort subite reste la cardiomyopathie hypertrophique (CMH) [1]. On peut citer aussi la cardiomyopathie dilatée et l’hypertrophie ventriculaire gauche comme autres cardiomyopathies entrainant une mort subite. C’est dire que pour les pays développés, la détermination de la cause de mort subite nécessitera au moins un examen macroscopique et un examen histologique et au mieux une étude toxicologique, génétique et immunohistochimique.

En Afrique et dans les pays en développement en général, la population est plus jeune que dans les pays développés. La population rurale est encore importante et l’urbanisation anarchique a entrainé l’émergence de mauvaises conditions socio-économiques et hygiéniques. La mortalité cardiovasculaire augmente au fil des années, alors que la mortalité générale diminue. On mange plus, mais on mange trop gras, trop sucré, trop salé, d’où l’apparition de facteurs de risque vasculaires. Les cardiopathies ischémiques et hypertensives augmentent alors que les cardiopathies rhumatismales ou nutritionnelles sont stables ou diminuent [11]. Les causes de mort cardiaque en Afrique sont l’hypertension artérielle, les valvulopathies rhumatismales et les cardiomyopathies infectieuses ou du post partum. Les complications seront les accidents vasculaires cérébraux, l’ischémie myocardique, les collapsus et les embolies pulmonaires [12], comme on le retrouve dans notre série. La cardiopathie rhumatismale reste présente et élevée dans la plupart des pays africains avec une prévalence de 25 à 30% [1, 13]. Au Sénégal on note une baisse de leur prévalence située entre 8 à 11% [12, 14]. Cette baisse des cardiopathies rhumatismales est liée à la prise en charge médicale correcte des angines de l’enfant par une antibiothérapie adaptée et prolongée systématique; cela explique le faible taux de valvulopathie dans notre série.

Les cardiomyopathies infectieuses sont un reflet des conditions sanitaires dépréciées par la promiscuité en ville favorisée par l’exode rural et un indicateur du niveau de médicalisation insuffisant du système sanitaire. Il en est de même des cardiomyopathies du post partum qui sont en plus liées à l’absence de médecins spécialistes dans les régions éloignées de la capitale.

Les morts subites de cause infectieuse surviennent après un choc septique lors d’infections fulminantes [15]. Aussi le Sénégal se trouvant en zone d’endémie palustre, le faible taux de morts subites par accès palustre dans notre série peut sembler anormal. Cela s’explique d’abord par le mode d’évolution prolongé de l’accès palustre qui entraine le décès par ses complications. Ensuite l’application des recommandations de l’OMS pour le traitement de tout accès fébrile par une association fixe à base d’artémisinine a induit une baisse de la mortalité liée au paludisme.

En somme, les causes de mort subite en Afrique seront essentiellement de diagnostic macroscopique à l’autopsie et accessoirement l’histologie complètera le diagnostic: on a plus de morts lésionnelles en Afrique. Pour les diagnostics les plus fréquents, la cause de mort est diagnostiquée dès l’examen macroscopique. C’est pourquoi dans notre série nous relevons 11,4% d’autopsies blanches.

## Conclusion

La mort subite, malgré les progrès de la médecine notamment les moyens d’investigation, reste fréquente avec plus de 24% des motifs d’autopsie dans notre série. Quatre causes principales sont retrouvées dans toutes les séries: cardiaques, pulmonaires, neurologiques et digestives. Bien que les résultats soient différents selon la sophistication de l’autopsie (utilisation de l’histologie, de la toxicologie), les causes cardiaques sont prépondérantes. L’autopsie reflète l’évolution de l’état de santé d’une population et constitue un indicateur utile au développement de politique de santé publique. Ces résultats doivent induire une politique visant à améliorer les conditions économiques et sociales des populations pour une maitrise des facteurs de risque vasculaire.
